# Clinical, radiological and pathological features of temporomesial tumors in the adult. A single center experience from 15 years

**DOI:** 10.3389/fonc.2023.1236269

**Published:** 2023-08-28

**Authors:** Hanno S. Meyer, Benedikt Wiestler, Lisa S. Hönikl, Claire Delbridge, Carl Ketterer, Jens Gempt, Bernhard Meyer

**Affiliations:** ^1^ Department of Neurosurgery, Klinikum rechts der Isar, School of Medicine, Technical University of Munich, Munich, Germany; ^2^ Department of Neurosurgery, University Medical Center Hamburg-Eppendorf, Hamburg, Germany; ^3^ Department of Neuroradiology, Klinikum rechts der Isar, School of Medicine, Technical University of Munich, Munich, Germany; ^4^ Center for Translational Cancer Research (TranslaTUM), School of Medicine, Technical University of Munich, Munich, Germany; ^5^ Department of Neuropathology, Klinikum rechts der Isar, School of Medicine, Technical University of Munich, Munich, Germany

**Keywords:** glioma, glioblastoma, temporal tumor, brain tumor, limbic encephalitis, autoimmune encephalitis

## Abstract

**Introduction:**

The mesial temporal lobe plays a distinct role in epileptogenesis, and tumors in this part of the brain potentially have specific clinical and radiological features. Differentiating high-grade from lower-grade tumors or non-neoplastic lesions can be challenging, preventing the decision for early resection that can be critical in high-grade tumors.

**Methods:**

A brain tumor database was analyzed retrospectively to identify patients with temporomesial tumors. We determined clinical features (age, sex, symptoms leading to clinical presentation) as well as neuroradiological (tumor location and the presence of contrast enhancement on initial magnetic resonance imaging (MRI)) and neuropathological findings.

**Results:**

We identified 324 temporal tumors. 39 involved the mesial temporal lobe. 77% of temporomesial tumors occured in males, and 77% presented with seizures, regardless of tumor type or grade. In patients 50 years or older, 90% were male and 80% had glioblastoma (GBM); there was no GBM in patients younger than 50 years. 50% of GBMs lacked contrast enhancement. Male sex was significantly associated with GBM. In both contrast-enhancing and non-enhancing tumors, age of 50 years or older was also significantly associated with GBM.

**Conclusion:**

In middle-aged and older patients with a mesial temporal lobe tumor, GBM is the most likely diagnosis even when there is no MRI contrast enhancement. Prolonged diagnostic workup or surveillance strategies should be avoided and early resection may be justified in these patients.

## 1 Introduction

Mass lesions affecting the temporal lobe are a frequent diagnostic and therapeutic challenge in neuro-oncology, neuroradiology and neurosurgery. They comprise not only neoplasms, but also vascular pathologies, inflammatory diseases, and developmental abnormalities. Differential diagnoses depend, among others, on age and clinical presentation. Temporal lesions are often recognized following epileptic seizures, especially if they are located in the mesial temporal lobe. They can also underly chronic epilepsy (1). In glioma patients, e.g., the majority of patients suffers from tumor-related epilepsy, with a higher prevalence in lower-grade gliomas compared to high-grade glioma (LGG/HGG); epilepsy may even be related to tumor growth (2, 3). Radiologically, it can be challenging to discriminate between high-grade and low-grade temporomesial tumors and non-neoplastic disease, such as paraneoplastic or virus-associated autoimmune encephalitis. Especially when magnetic resonance imaging (MRI) shows an expansive lesion with hyperintense signal in T2-weighted (T2w) and T2-fluid-attenuated inversion recovery (FLAIR) imaging that is not contrast-enhancing, it is often difficult to distinguish limbic autoimmune encephalitis from LGG or HGG.

In both LGG and HGG, standard therapy entails maximal safe tumor resection; this may be followed by observation in specific constellations, but is usually succeeded by standardized adjuvant radiation and chemotherapy regimes (4–11). In specific cases, novel individualized therapies such as AI-based radiotherapy planning or patient-tailored immunochemotherapy may be an option (12). HGG demands prompt resection surgery in order to completely remove a potentially rapidly growing tumor before it becomes unresectable, whereas LGG permits more time and autoimmune encephalitis is not treated surgically. Consequently, radiological findings are critical for clinical decision making, e.g., when deciding whether to opt for prompt resection surgery or to initiate further diagnostics or medical treatment attempts that might delay time-critical surgical treatment.

In this study, we analyzed clinical and radiological features of patients with mesial temporal lobe tumors in order to investigate whether prolonged diagnostic workup or even a wait and see strategy is justified in patients with non-contrast-enhancing lesions.

## 2 Materials and methods

### 2.1 Study design, patient selection

We retrospectively analyzed a prospectively collected database of 1265 patients who were treated for a brain tumor at the Department of Neurosurgery at the Klinikum rechts der Isar of the Technical University of Munich in the past 15 years. Surgery for a brain tumor at our institution was the only inclusion criterion; no patient was excluded from the analysis. The database comprises demographic and clinical data including sex, age at surgery, and clinical presentation, information on different treatment modalities, radiological data, and pathology findings. All temporal tumors were reviewed to identify patients with lesions involving the mesial temporal lobe. It is important to note that our center almost exclusively treats adult patients. Moreover, the indication for surgery was based on the recommendation of an interdisciplinary tumor board, and whenever non-neoplastic diseases were considered as possible differential diagnosis, patients underwent comprehensive neurological workup to rule out, e.g., virus-mediated/autoimmune encephalitis or other inflammatory diseases based on a close cooperation with our neurology department. In these cases, surgery was recommended only when a non-neoplastic disease could not be confirmed.

### 2.2 Imaging

All patients received preoperative MR imaging, including the acquisition of contrast-enhanced T1-weighted (T1w) sequences. The minimum imaging protocol available for all patients included a T2w-/FLAIR sequence as well as T1w images before and after administration of contrast agent. In addition, diffusion- and perfusion-weighted imaging (dynamic susceptibility contrast) was available for some patients. Image analysis comprised the assessment of tumor shape, solidity, infiltration and contrast-enhancement. Where available, ADC (Apparent diffusion coefficient; from diffusion-weighted imaging) and CBV maps (Cerebral blood volume; from perfusion-weighted imaging) were additionally investigated. Some patients received preoperative O-(2-[^18^F]Fluoroethyl)-L-tyrosine PET imaging. PET scans were obtained using a Biograph 16 PET/CT or a Biograph mMR PET/MRI (both from Siemens Medical Solutions USA, Malvern, PA, USA).

### 2.3 Treatment

Patients underwent microsurgical resection aiming at maximal safe tumor removal based on the recommendation by an interdisciplinary tumor board. Standard procedure at our institution includes transcranial magnetic stimulation – based preoperative functional language and motor mapping as well as intraoperative navigation and neuromonitoring. Post-resection adjuvant treatment was based on tumor board recommendations and included radiation and/or chemotherapy if indicated according to current treatment standards (4–11).

### 2.4 Neuropathology

All specimen were formalin-fixed and paraffin embedded. After histological assessment, immunohistochemistry with standard diagnostic markers was performed. Depending on the year of operation, the respective state of the art diagnostics according to the then current WHO classification were applied (13–15). All specimen were evaluated by two board certified neuropathologists. Isocitrate dehydrogenase (IDH) mutation and 1p/19q codeletion were assessed using immunohistochemistry for IDH1 (R132H) and Sanger sequencing (16). Starting in 2020, all tumors were classified using 850K methylation assay (17). Diagnoses are reported according to the latest WHO classification (2021); accordingly, in cases lacking all necessary molecular information, the original diagnosis is given but labeled as “not otherwise specified” (NOS). All GBMs were IDH wildtype.

### 2.5 Statistical analysis

Statistical analyses were carried out using IBM SPSS statistics software (version 29.0.0.0).

## 3 Results

324 out of 1265 patients in our brain tumor database had a tumor that involved the temporal lobe. Our center almost exclusively treats adult patients, which is reflected in the fact that 317 (98%) of these were 18 years or older. The median age at surgery was 58 years and the interquartile range (IQR) was 46-71 years. 195 were male, corresponding to a male-to-female ratio of 1.5:1.

39 of the temporal tumors affected the mesial temporal lobe. Except for two patients aged 10 and 15 years, all were adults. With a median age at surgery of 50 years (IQR: 33-61 years), patients with temporomesial tumors were significantly younger than patients with other temporal tumors (median age: 59 years; IQR: 48-72 years; p=0.0007, two-tailed t-test). The male-to-female ratio was markedly higher in mesial temporal lobe tumors than in other temporal tumors (3.3 vs. 1.4) and there was a significant association between male sex and mesial temporal tumor location in patients with temporal tumors (p=0.023, chi-square test).

**Figure 1:**
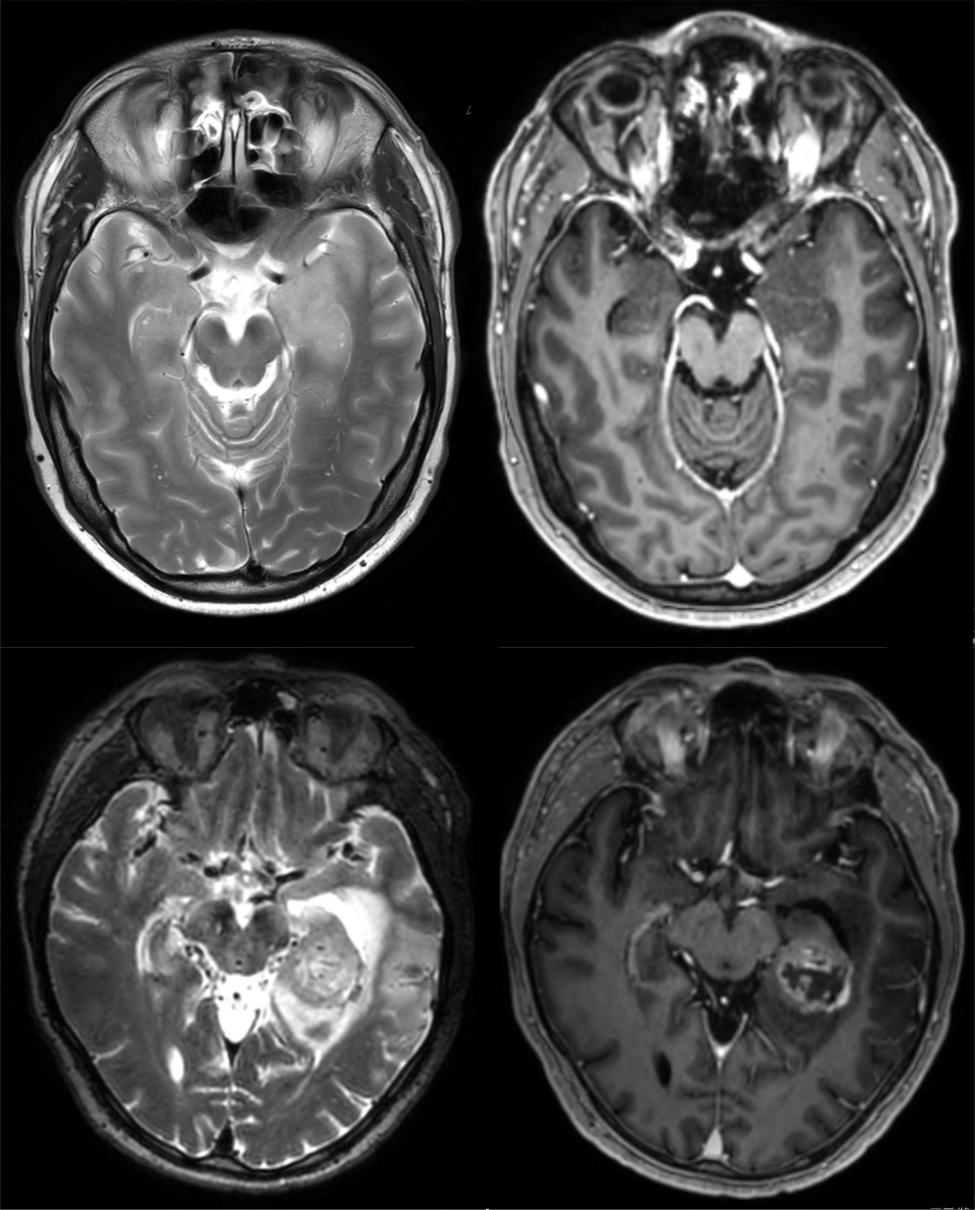
Illustration of contrast-enhancing and non-contrast-enhancing mesial temporal lobe tumors. MRI images from a patient with a non-contrast-enhancing tumor (upper panels) are shown in comparison with MRI images from a patient with a contrast-enhancing tumor (lower panels). Upper left panel: T2-weighted MRI showing a mass lesion in the left anterior mesial temporal lobe. Upper right panel: Corresponding T1-weighted MRI (post-gadolinium), showing that there is no contrast-enhancement within the lesion. Lower left panel: a different patient with a mass lesion in the left hippocampus as seen on T2-weighted MRI. Lower right panel: Different from the other case, this lesion is contrast-enhancing, indicating disruption of the blood brain barrier and consistent with a high-grade glioma. PET imaging for the two cases is shown in **Figure 2**.

**Figure 1** shows MRI examples of patients with mesial temporal lobe tumors. **Table 1** illustrates demographic as well as radiological, clinical and pathological features of patients with temporomesial tumors. Pathological diagnoses are reported consistent with the 2021 WHO classification of tumors. Accordingly, in cases without sufficient molecular data, the original diagnosis is used and labeled as “not otherwise specified” (NOS).

**Table 1:** Clinical, radiological and pathological features of patients with a mesial temporal lobe tumor.

C.E.^1^	Age [Years]	Pts. [n]	Male [n/%]	Seizure [n/%]	Diffuse Gliomas				GN [n/%]
					GBM, IDH wt [n/%]	Astro, IDH mut [n/%]	Oligo, 1p/19q codel [n/%]	Glioma, NOS [n/%]	
Yes	<50	3	2 (67%)	2 (67%)	0	0	1 (33%)	0	2 (67%)
	≥50	8	7 (88%)	6 (75%)	8 (100%)	0	0	0	0
No	<50	16	10 (63%)	12 (75%)	0	1 (6%)	2 (13%)	8 (50%)	5 (31%)
	50-60	8	8 (100%)	8 (100%)	8 (100%)	0	0	0	0
	>60	4	3 (75%)	2 (50%)	0	0	0	2 (50%)	2 (50%)

^1^ C.E.: Tumor with contrast enhancement on initial MRI. Pts.: Number of patients in subgroup. Percentages refer to all patients within subgroup, respectively. GBM, IDH wt: glioblastoma, IDH wild type, WHO grade 4. Astro, IDH mut: astrocytoma, IDH mutated. Oliqo, 1p/19q codel: oligodendroglioma, 1p/19q co-deleted. NOS: Diffuse glioma, not otherwise specified. GN, glioneuronal and neuronal tumors. All GBMs were IDH wildtype. All GNs had WHO grade 1.

Male patient sex was significantly associated with the final diagnosis of GBM (p=0.0375, chi-square test).

11 patients had tumors with contrast enhancement on MRI. In this group, all patients older than 50 years had a glioblastoma (GBM, IDH wildtype, WHO grade 4). In fact, patient age of 50 years or older was significantly associated with the diagnosis of a GBM (p=0.0009, chi-square test) in patients with contrast-enhancing lesions. Almost all of these patients were male (seven out of eight) and had epileptic seizures (six out of eight). In younger adults with contrast-enhancing tumors, there was no GBM (one ganglioglioma grade 1, one gangliocytoma grade 1, and one anaplastic oligodendroglioma, 1p/19q co-deleted, grade 3).

The larger group of patients harbored tumors without contrast enhancement (n=28). In this group, patient age of 50 years or older was also significantly associated with the diagnosis of a GBM (p=0.0001, chi-square test). Surprisingly, there was a homogeneous subgroup of non-contrast-enhancing temporomesial tumors: all patients aged 50 to 60 years were male, presented with seizures, and had a GBM, IDH wildtype. On the other hand, non-contrast-enhancing tumors in older patients (>60 years) were a much more heterogeneous group, with two glioneuronal tumors (both grade 1) and two diffuse gliomas (one anaplastic oligodendroglioma, NOS, grade 3 and one anaplastic astrocytoma, NOS, grade 3), and only 50% had seizures. As in non-enhancing tumors, there was no GBM in patients younger than 50 years, a subgroup of 10 male and 6 female patients that comprised five glioneuronal/neuronal grade 1 tumors (four gangliogliomas and one angiocentric glioma) and 11 diffuse gliomas of lower grade (two oligodendrogliomas, IDH mutated and 1p/19q co-deleted, grade 2; one astrocytoma, IDH mutated, grade 2; five astrocytomas, NOS, grade 2, and three anaplastic astrocytomas, NOS, grade 3). 75% of these patients presented with epileptic seizures.

**Figure 2:**
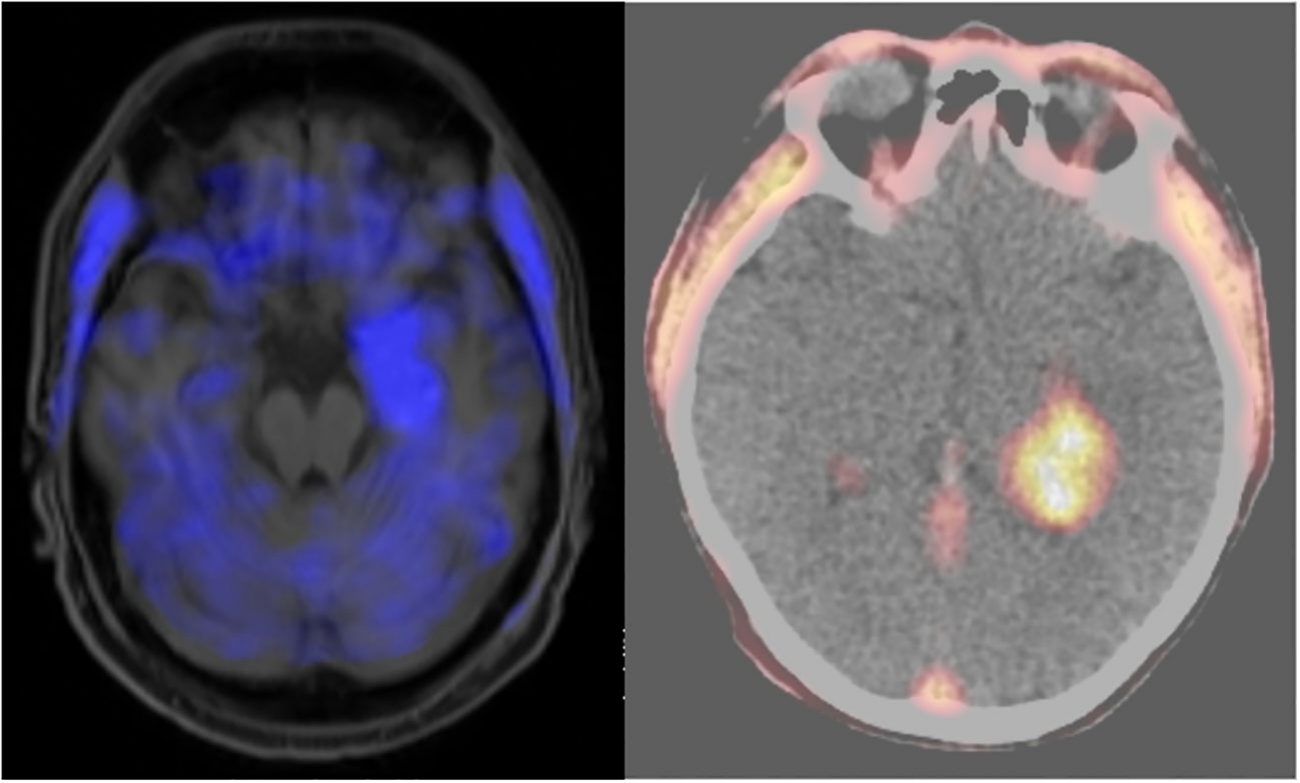
18F FET PET imaging from to the two cases shown in **Figure 1**. Left: PET MRI image corresponding to the MRI imaging planes shown in the upper panels of **Figure 1**. There was slightly positive tracer uptake in the non-MRI contrast-enhancing tumor (the mean tumor-to-background ratio (TBR) was 1.6, with a normal TBR being <1.6), indicating a possible high-grade tumor. Right: PET CT image corresponding to the MRI imaging planes shown in the lower panels of **Figure 1**. There is high tracer uptake in the MRI contrast-enhancing tumor, supporting the suspicion of a high-grade tumor (TBR = 3.1). Both patients had glioblastoma.

To further investigate the properties of non-contrast enhancing temporomesial GBM in middle aged males, we searched for additional clinical and diagnostic information. For six of these eight patients, FET PET imaging was available (**Figure 2**). We found that two of them (33%) were PET positive (i.e., suggestive of a high-grade glioma). MGMT promotor methylation was present in two out of seven cases with available data (28.6%). Of note, three patients had initially been treated neurologically based on an unconfirmed suspicion of limbic encephalitis for several weeks. The tumors of two patients progressed and developed contrast enhancement before they had surgery within several days. In primarily contrast-enhancing temporomesial GBM, on the other hand, all three available PETs were positive (100%), and MGMT promotor methylation was present in three out of six cases with available data (50%). None of these cases was initially suspected to be encephalitis.

## 4 Discussion

We found that seizures were the presenting symptom in more than three out of four patients with a tumor in the mesial temporal lobe, which was true for patients with higher and lower grade tumors (grades 3, 4: 77%; grades 1, 2: 77%). For higher grade tumors, this is more frequent than expected: in previous studies that did not take tumor location into account, seizure frequencies ranging from 20-40% have been reported (18, 19).

The fact that both low-grade and high-grade tumors of the mesial temporal lobe frequently present with seizures might appear to contradict the notion that low-grade tumors are more epileptogenic than high-grade tumors (1, 20–23). However, while this observation refers to long-term epilepsy-associated tumors (24–26), it is important to point out that our patients did not suffer from chronic epilepsy, but typically presented with single or a few novel focal or generalized seizures that led to the diagnosis.

The high frequency of seizures being the initial clinical manifestation in different types of mesial temporal lobe tumors can be explained by the eminent role of mesial temporal lobe structures in epileptogenesis. This might be the reason why in our series, patients with mesial temporal lobe tumors are younger than patients with tumors in other parts of the temporal lobe, as the former become clinically apparent earlier than the latter. Finally, this may also underly the remarkable finding that 50% of glioblastomas were non-contrast-enhancing: It can be speculated that these have been imaged very early in the course of the disease and would not have become symptomatic so early if they had grown in less epileptogenic parts of the brain.

Most importantly, our data underlines that a “wait and see” approach or even a prolonged diagnostic workup and/or treatment for limbic encephalitis may not be justified when facing a non-enhancing mass involving the mesial temporal lobe in middle-aged patients, as it is likely to represent a GBM. Delayed surgery harbors the risk for incomplete resections, as illustrated by cases that had tumor progression in our series.

FET PET can potentially support the decision for resection surgery in these situations: in 33%, it indicated a HGG despite a lack of MRI contrast enhancement. However, this might also represent tumor progression rather than higher sensitivity, and further studies are required to clarify whether FET PET can detect HGG earlier than MRI.

Interestingly, we found that a tumor of the mesial temporal lobe is a predominantly male disease. While it is known that the incidence of both IDH-mutant gliomas and GBM is higher in males than in females at an approximate ratio of 1.3:1 (27), especially older males appear to be even more prone to gliomas of the mesial temporal lobe: in our series, the male-to-female ratio was 3.3:1, and it increased to 9:1 in patients older than 50 years.

We describe a remarkably clinically well-defined subgroup of patients with tumors of the mesial temporal lobe. In non-contrast-enhancing tumors, all patients aged 50 to 60 years were male, presented with seizures, and turned out to have GBM. Future prospective studies will investigate whether common molecular, genetic or epigenetic features underly this clinical homogeneity and whether this descriptive characterization based on a rather arbitrary age cutoff actually corresponds to a distinct entity.

Our study has limitations. Most importantly, this was a retrospective analysis, potentially introducing bias. Importantly, there is selection bias based on the indication for surgery as confirmed by the interdisciplinary tumor board for all cases in our database: Our cohort includes cases with clear suspicion for a tumor by means of imaging (e.g., irregularly contrast-enhancing mass lesion, lower panels in **Figure 1**), but it also includes cases with less clear imaging findings (e.g., subtle enhancement on T2-weighted MRI, no contrast-enhancement, upper panels in **Figure 1**) provided that a comprehensive neurological workup did not confirm non-neoplastic disease (see Methods/patient selection). It is also important to note that even though we investigated a large cohort, only a relatively small number of patients had temporomesial tumors, and the homogeneous subgroup of middle-aged male GBM patients with non-enhancing tumors is even smaller. This means that further study on this matter is warranted to validate our findings in larger cohorts of patients. Moreover, given the long period of time covered by our database, state-of-the-art molecular pathology data was not available for all cases. While the 3^rd^ edition of 2007 mainly focused on histomorphological criteria (13), the 4^th^ edition of the WHO classification (2016) introduced molecular markers (14). The combined chromosomal loss of 1p and 19q defined an oligodendroglioma then, but an IDH mutation did not yet separate GBM from astrocytoma or oligodendroglioma, leading to the diagnosis of GBM, IDH-mutated or IDH wildtype. The latest version of the WHO classification (2021) sharply separates GBM, IDH wildtype from high-grade astrocytoma, IDH-mutated or oligodendroglioma, IDH-mutated (15). This has to be taken into account when evaluating and comparing tumor diagnosis in this cohort that spans 15 years. However, high-grade gliomas were separated very clearly from low-grade tumors in this series, and we confirmed that all glioblastomas in this cohort were IDH wildtype.

## 5 Conclusions

Tumors of the mesial temporal lobe are clinically and radiologically distinct. Seizures are common in all tumor grades, and most patients are male, especially those 50 years or older. Apparently, in terms of MRI, not all GBMs glitter: in mesial temporal lobe tumors, lack of MRI contrast enhancement does not rule out high grade glioma at all. Rather, most middle-aged or older patients with non-contrast enhancing lesions and without limbic/autoimmune encephalitis turn out to suffer from GBM, prohibiting prolonged surveillance and warranting early resection.

## Data availability statement

The raw data supporting the conclusions of this article will be made available by the authors, without undue reservation.

## Ethics statement

The studies involving humans were approved by the Ethics Committee of the Klinikum rechts der Isar of the Technical University of Munich (5625/12). The studies were conducted in accordance with the local legislation and institutional requirements. The ethics committee/institutional review board waived the requirement of written informed consent for participation from the participants or the participants’ legal guardians/next of kin because this was a retrospective analysis of anonymized data.

## Author contributions

Conceptualization, BM, HM and BW. Methodology, HM and BW. Formal analysis, HM and BW. Investigation, HM, BW, LH, CK, CD and BM. Data curation, LH, HM, CK and JG. Writing—original draft preparation, HM. Writing—review and editing, BW, CD and BM. Visualization, BW. Supervision, BM. All authors contributed to the article and approved the submitted version.
